# Wireless, non-invasive, wearable device for continuous remote monitoring of hemodynamic parameters in a swine model of controlled hemorrhagic shock

**DOI:** 10.1038/s41598-020-74686-6

**Published:** 2020-10-19

**Authors:** Dean Nachman, Keren Constantini, Gal Poris, Linn Wagnert-Avraham, S. David Gertz, Romi Littman, Eli Kabakov, Arik Eisenkraft, Yftach Gepner

**Affiliations:** 1 Institute for Research in Military Medicine, Faculty of Medicine, The Hebrew University of Jerusalem and the Israel Defense Force Medical Corps, Ein-Kerem Campus, POB 12272, 9112001 Jerusalem, Israel; 2 Heart Institute, Hadassah Ein Kerem Medical Center, POB 12000, 9112001 Jerusalem, Israel; 3 Department of Epidemiology and Preventive Medicine, School of Public Health, Sackler Faculty of Medicine and Sylvan Adams Sports Institute, Tel Aviv University, Tel Aviv, Israel; 4 Biobeat Technologies LTD, 26 Magshimim Street, Petah Tikva, Israel

**Keywords:** Biotechnology, Physiology, Biomarkers, Cardiology, Diseases, Health care, Medical research, Signs and symptoms

## Abstract

Accurate and continuous monitoring of critically ill patients is frequently achieved using invasive catheters, which is technically complex. Our purpose was to evaluate the validity and accuracy of a photoplethysmography (PPG)-based remote monitoring device compared to invasive methods of arterial line (AL) and Swan-Ganz (SG) catheters in a swine model of controlled hemorrhagic shock. Following a baseline phase, hemorrhagic shock was induced in 11 pigs by bleeding 35% of their blood volume, followed by a post-bleeding follow-up phase. Animals were monitored concomitantly by the PPG device, an AL and a SG catheter, for a median period of 447 min. Heart rate (HR), systolic and diastolic blood pressure (SBP and DBP, respectively), and cardiac output (CO) were recorded continuously. The complete data set consisted of 1312 paired observations. Correlations between the PPG-based technique and the invasive methods were significant (*p* < 0.001) during baseline, bleeding and follow-up phases for HR (*r* = 0.90–0.98), SBP (*r* = 0.90–0.94), DBP (*r* = 0.89–0.93), and CO (*r* = 0.76–0.90). Intraclass correlations for all phases combined were 0.96, 0.92, 0.93 and 0.87 for HR, SBP, DBP and CO, respectively. Correlations for changes in CO, SBP and DBP were significant (*p* < 0.001) and strong (*r* > 0.88), with concordance rates (determined by quadrant plots) of 86%, 66% and 68%, respectively. The novel PPG-based device was accurate and valid compared to existing invasive techniques and might be used for continuous monitoring in several clinical settings following further studies.

## Introduction

Monitoring of cardiovascular parameters, including heart rate (HR), blood pressure (BP), and cardiac output (CO), plays a vital role in the assessment, treatment, and follow-up of patients with different medical conditions^1,2^. Although trauma and resuscitation protocols have improved over the years, systemic organ failure still occurs in a relatively high percentage of patients suffering from acute and chronic life-threatening diseases^3–8^. Aggressive resuscitation using accurate cardiovascular monitoring can reduce tissue hypoxia, attenuate oxidative damage, prevent progression to multi-organ failure, and improve outcome in these clinical scenarios^9^. Ideally, the technology for measuring cardiovascular parameters should allow continuous, non-invasive, wireless, safe, inexpensive monitoring, easy to operate remotely, while being accurate and reliable.

Over the past years, development efforts focused on methods that fulfil these requirements. Currently, the gold standard for hemodynamic monitoring of critically ill patients includes the insertion of an arterial line (AL) to measure BP and HR, and a Swan-Ganz (SG) pulmonary artery catheter to measure CO. These methods are relatively accurate and reliable, but invasive, with the potential of adverse events such as infection and damage to the arteries through which they were inserted. In addition, large retrospective and randomized controlled trials demonstrated no improvement in the mortality of patients following the use of these invasive methods compared to patients without invasive monitoring^10–12^. Yet, some studies have shown that use of a pre-emptive strategy of hemodynamic monitoring including HR, BP and CO reduces morbidity and surgical mortality with the advantages and precision of the invasive measurement methods considered to outweigh their complications^9,13,14^. On the other hand, although any medical intervention has associated risks, it has been noted in several clinical studies that the number of life-threatening complications associated with the SG catheter is relatively high^15^.

In this study, we evaluated the monitoring capability of a new device (Biobeat Technologies LTD, Petah-Tikva, Israel), which measures cardiovascular parameters using a reflective photoplethysmography (PPG) sensor. This sensor allows non-invasive, cuff-less, and wireless measurements of HR, systolic blood pressure (SBP), diastolic blood pressure (DBP), and CO. The accuracy and validity of the device were determined by head-to-head comparison with readings from an AL and a SG catheter in a swine model of controlled hemorrhagic shock, an accepted model to study trauma care as described later.

## Results

During all study phases, SG and AL, as well as the wearable device, provided continuous assessment of physiological parameters. Two animals did not undergo a bleeding phase due to early systemic decompensation, and therefore only baseline (i.e., pre-bleeding) data is reported for those animals. Details about the blood volume removed from each animal, and clinical data recorded during the study are presented in Table 1. In general, we did not see any major clinical event in any of the animals during the study. The number of observations (total of 1306) and mean ± SD values for each variable per stage are presented in Table 2.

**Table 1:** Clinical data of animals included in the study.

Animal No	Weight (kg)	Blood volume removed (ml)	Events of clinical importance during the post-bleeding period
1	47.5	1100 (35% within 30 min)	Started the protocol with 39.9 °C, 20 min after completion of bleeding phase showed tachycardia, 50 min after completion of bleeding abdominal distension was apparent. Completed 7 h of observation
2	56	1300 (35% within 20 min)	Completed 7 h of observation
3	47.5	1120 (35% within 55 min)	Ventricular arrhythmias appeared 5 h 40 min after completion of the bleeding phase, leading to VF and death 10 min later, at 5 h50 min after completion of bleeding
4	47	1050 (33% within 1 h)	Completed 7 h of observation
5	48	1125 (35% within 25 min)	Multi-organ failure and rapid death with no prior hemodynamic changes, 2 h after completion of bleeding phase
6	46	1100 (35% within 35 min)	Completed 7 h of observation
7	68	None	
8	55	1295 (35% within 40 min)	Completed 7 h of observation
9	45	900 (30% within 1 h)	Completed 7 h of observation
10	50	None	
11	50	1175 (35% within 1 h)	Completed 7 h of observation

**Table 2:** Number of observations and mean ± SD values for each variable per stage.

Variable	Phase	N	Mean ± SD	
			Invasive	BB-613WP
SBP (mmHg)	Pre-bleeding	128	79 ± 14	81 ± 15
	Bleeding	103	51 ± 20	58 ± 20
	Post-bleeding	106	58 ± 12	62 ± 10
DBP (mmHg)	Pre-bleeding	128	57 ± 18	59 ± 17
	Bleeding	103	32 ± 12	38 ± 12
	Post-bleeding	106	30 ± 18	35 ± 18
HR (BPM)	Pre-bleeding	128	96 ± 20	95 ± 14
	Bleeding	103	99 ± 20	99 ± 19
	Post-bleeding	104	125 ± 25	124 ± 26
CO (l/min)	Pre-bleeding	128	3.8 ± 1.0	4.2 ± 0.9
	Bleeding	91	2.4 ± 1.1	3.0 ± 1.1
	Post-bleeding	78	2.2 ± 0.5	2.7 ± 0.6

*SBP* systolic blood pressure, *DBP* diastolic blood pressure, *HR* heart rate, *CO* cardiac output.

### Degree of agreement

**Figure 1:**
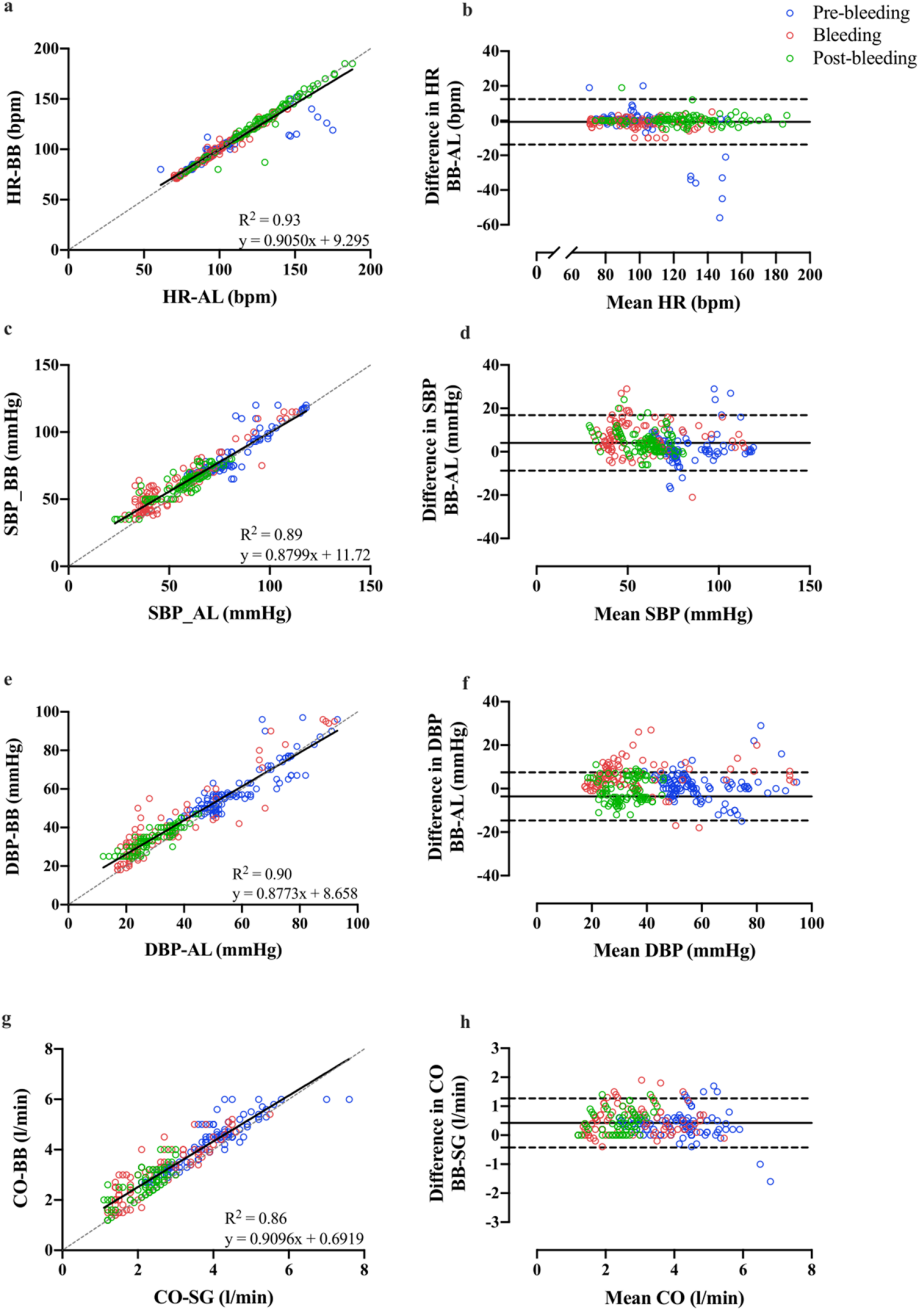
Bland–Altman plots of the relationship (right panels) and agreement (left panels) between the PPG-based device and invasive measures. Measurements obtained by the PPG-based device, arterial line (AL) or Swan-Ganz (SG) include heart rate [HR; (**a**,**b**)], systolic blood pressure [SBP; (**c**,**d**)], diastolic blood pressure [SBP; (**e**,**f**)], and cardiac output [CO; (**g**,**h**)]. In the left panels, the solid line is the best fit linear regression and the dash line is the line of identity. In the right panels, the solid horizontal line represents the mean difference between the two measurements and the dash horizontal lines represent the limits of agreement. Blue symbols: pre bleeding phase; red symbols: bleeding phase; green symbols: post bleeding phase.

**Figure 2:**
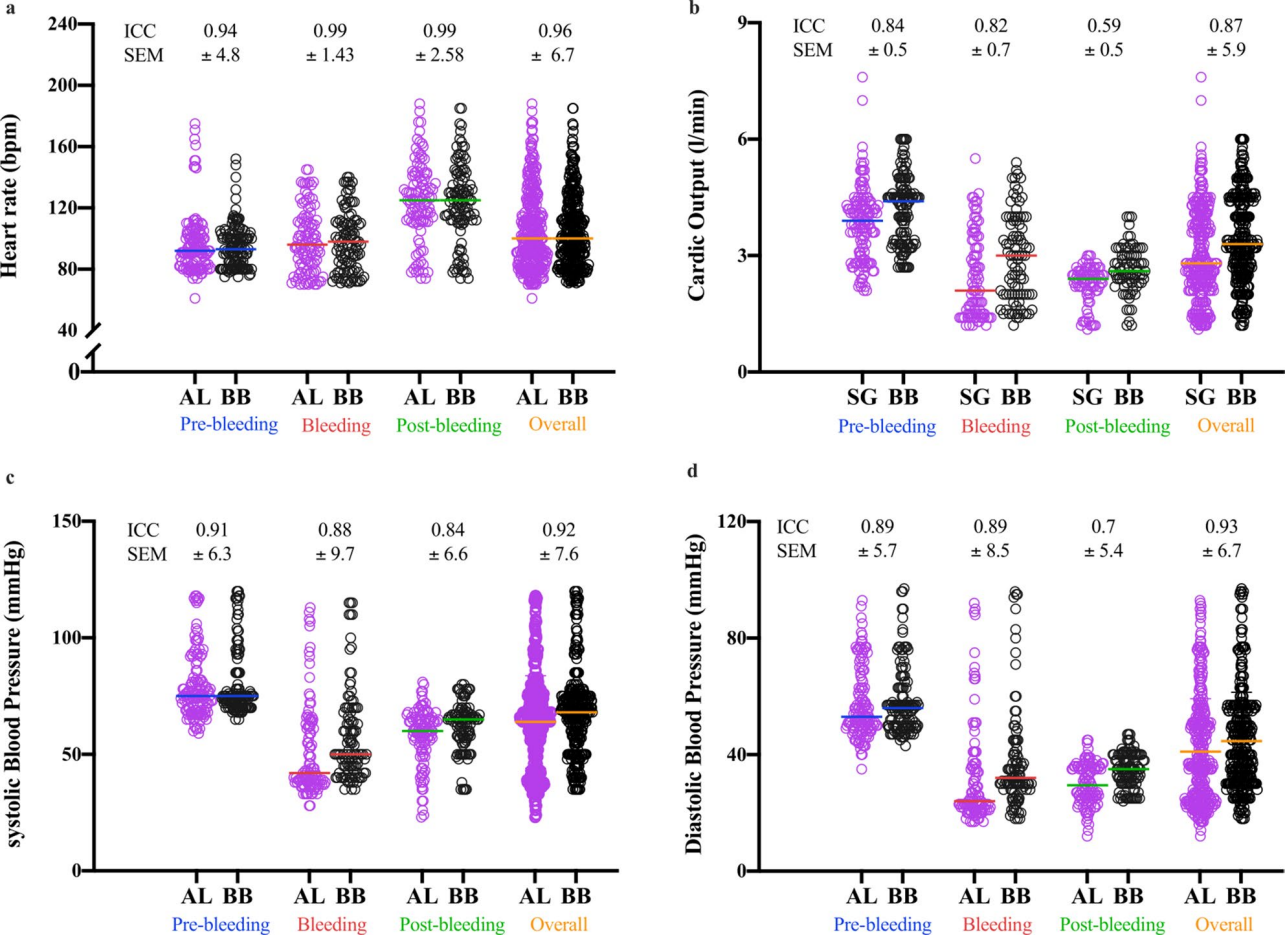
Individual values, variability, and agreement between the PPG-based device and invasive measures across study phases. Values are shown for heart rate (**a**), cardiac output (**b**), systolic blood pressure (**c**) and diastolic blood pressure (**d**). *BB* PPG-based device, *AL* arterial line, *SG* Swan Ganz, *ICC* intraclass correlation coefficient, *SEM* standard error of the mean. Colored horizontal lines represent the median.

Pearson correlations and Bland–Altman plots with 95% limits of agreement (LOA) for each variable are presented in Fig. 1. HR values obtained by the AL (107 ± 28 bpm) and the PPG-based device (106 ± 27 bpm) were significantly (*p* < 0.001) and strongly correlated during all phase (pre-bleeding: *r* = 0.90; bleeding: *r* = 0.99; post-bleeding: *r* = 0.98). Overall mean values for the invasive measurement were 65 ± 25 and 42 ± 24 mmHg for SBP and DBP, respectively. For the PPG-based device, SBP was 69 ± 24 mmHg and DBP was 46 ± 23 mmHg. There also were significant (*p* < 0.001) and strong correlations between methods of measurement of BP during all phases (SBP: pre-bleeding: *r* = 0.91; bleeding: *r* = 0.94; post-bleeding: *r* = 0.90, and DBP: pre-bleeding: *r* = 0.89; bleeding: *r* = 0.93; post-bleeding: *r* = 0.90). CO values from SG (4.0 ± 17.1 l min^−1^) and the wearable device (4.4 ± 17.0 l min^−1^) were significantly (*p* < 0.001) correlated during each phase (pre-bleeding: *r* = 0.90; bleeding: *r* = 0.90; post-bleeding: *r* = 0.76). Intraclass correlation coefficients (ICC) and standard error of the mean (SEM) per phase between each pair of measurements have been tested and presented in Fig. 2. For all phases combined, ICC (± SEM) was 0.96 (6.7), 0.92 (7.6), 0.93 (6.7), and 0.87 (5.9) for HR, SBP, DBO, and CO, respectively.

**Figure 3:**
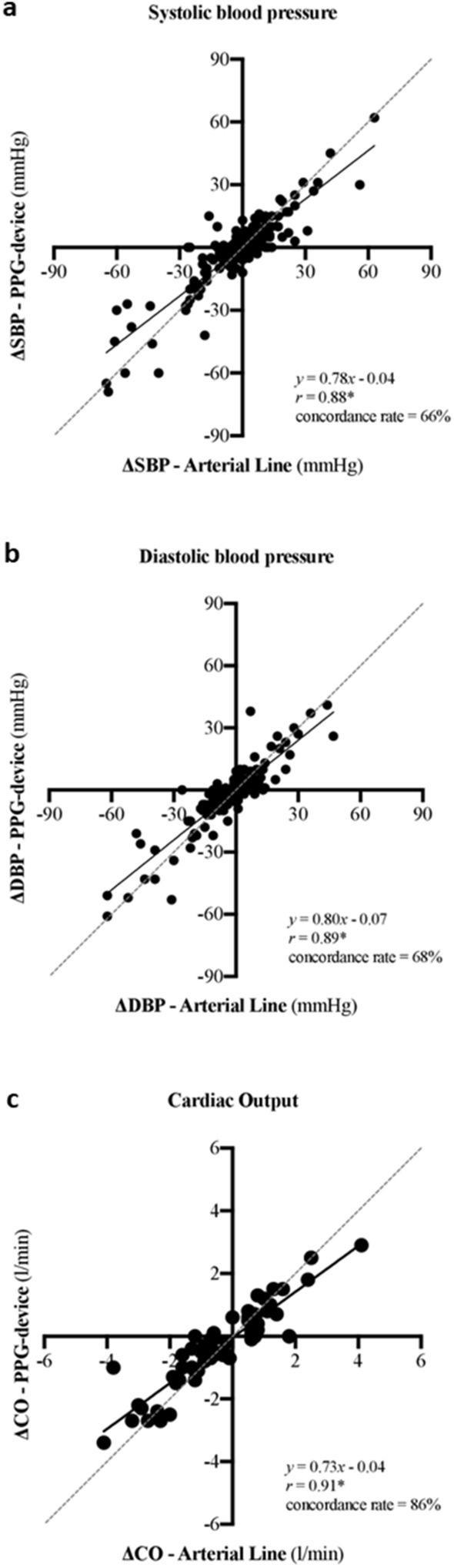
Four quadrant plots for (**a**) systolic blood pressure (SBP), (**b**) diastolic blood pressure (DBP) and (**c**) cardiac output (CO). These analyses investigate trending abilities and represent the level of agreement between and concordance rate of successive changes in the photoplethysmography-based device and invasive techniques (i.e. arterial line for blood pressure and Swan-Ganz for CO). *p < 0.001.

Correlations between AL/SG and the PPG-based device for changes in SBP, DBP and CO were significant (*p* < 0.001) and strong (*r* > 0.88 for all; Fig. 3). The concordance rates, determined by four quadrant plots, were 66%, 68% and 86% for SBP, DBP and CO, respectively.

## Discussion

Invasive monitoring methods provide continuous measurement of numerous cardio-pulmonary parameters, yet they are prone to complications that could endanger patients’ lives^10,16^. Thus, advanced hemodynamic monitoring by non-invasive means might enable thoughtful diagnosis and treatment of unstable patients while minimizing morbidity and mortality. In this work, we have shown that the PPG-based device provides accurate and valid readings with marginal bias and narrow LOAs for SBP, DBP, HR, and CO when compared to the invasive, gold-standard measurements obtained from AL and SG catheters. Our findings demonstrated a high level of correlation between the techniques during all stages of the study, including during dynamic and substantial fluctuations that occurred throughout the bleeding and post-bleeding phases. These phases are characterized by high hemodynamic variability and low reading inputs, suggesting that the wearable device is valid for use under unstable physiological conditions.

In the past, there have been several commercial and academic efforts to develop non-invasive, advanced hemodynamic monitoring technologies^17–19^. As of today, none of these techniques have gained widespread acceptance or use due to conflicting validation data and cumbersome implementation of the instrumentation^20,21^. PPG is commonly-used for non-invasive monitoring of arterial oxyhemoglobin saturation and HR in research and applied settings; however, several studies have shown that the waveform produced is affected by systemic circulation changes^22,23^, and the accuracy and reliability of data obtained from PPG-based devices remain questionable, particularly under dynamic conditions such as in hemodynamic shock. Nevertheless, results from this study show that the higher temporal resolution of the PPG sensor we used allows accurate detection of PPG waves even during periods of hemodynamic instability. The advanced algorithms applied to the pulse contour readings by the device allow for enhanced accuracy and reliability of HR, SBP, DBP, and CO monitoring (Fig. 1).

Although in many situations BP is measured non-invasively (i.e. without the insertion of an AL) and by automated devices, the necessity of using a cuff makes this measurement cumbersome and inconvenient for both patients and care providers, particularly in circumstances where beat-to-beat measurements are required or in isolated patients. While criteria exist for assessing the validity of automatic BP devices and acquiring approval by the European Society of Hypertension (ESH), American Heart Association, Food and Drug Administration and other governing bodies, criteria for cuff-less monitors such as the sensor used in this study have yet to reach widespread, general acceptance. Based on the ESH’s International Protocol for the validation of BP measuring devices^24^, post-hoc analysis revealed as many as 93% of the SBP values obtained from the wearable device were within ± 15 mmHg of those recorded by AL. A similar analysis for DBP yielded even more remarkable results with 97% of the wearable device’s values falling within ± 15 mmHg of AL. Furthermore, we have shown high levels of agreement (Pearson’s correlation > 0.96 and ICC’s > 0.92 for both SBP and DBP; Figs. 1 and 2), and relatively narrow 95% LOAs (SBP: − 9, 17; DBP: − 8, 15) between the PPG-based device and the AL measurements, suggesting the wearable device can accurately and reliably measure SBP and DBP under unstable hemodynamic conditions.

PPG-based devices for the measurement of HR have become widely used in various settings despite some reports of questionable accuracy and reliability^18,25,26^. A recent study investigating the agreement between electrocardiogram and PPG-based HR measurements reported an ICC of 0.90 and mean difference between devices of 2.7 beat/min^−1^^27^. While these findings may appear promising, it should be mentioned that the overall ICC for HR in this study was 0.96, the mean difference between techniques was < 1 beat/min^1^ (Figs. 1, 2), and 85% of non-invasive measurements were within 3 beat/min^1^ of AL-derived HR^28^. Identifying accurate HR and BP readings, particularly under unstable hemodynamic conditions, is fundamental in providing optimal medical treatment and improving patient outcomes and prognosis. Therefore, the high level of accuracy and strong agreement between the wearable device and the AL measurements for HR and BP under dynamic physiological conditions suggests the need for testing this device in humans.

Of the hemodynamic variables measured in this study that are relevant and important for clinically unstable patients, CO is the most challenging one to assess accurately using non-invasive methods. As mentioned above, although various attempts have been made to develop techniques for minimal or non-invasive measurement of CO, the accuracy and reliability of these methods have been inconsistent and therefore unsatisfactory for clinical use at this time^17,19,21,29,30^. For example, while the clinically acceptable percentage error (i.e. LOA as a proportion of mean CO) for CO monitors is as high as 30%^31,32^, most devices have an error of ~ 40%^19,29^, making them unacceptable for clinical use. To the best of our knowledge this is the first scientific study to compare a high-resolution PPG technique with gold standard invasive measurements of CO. We have shown that the percentage error of the wearable device was as low as 25%, with a small bias (0.42 l/min^−1^) and relatively narrow LOA (Fig. 1) compared to SG in a swine model of controlled hemorrhagic shock.

When analyses were performed per phase, intraclass and Pearson correlations between the SG and the PPG-based device for the pre-bleeding and bleeding phases were relatively high, while post-bleeding correlation analyses were not as strong (Fig. 1). It should be emphasized that due to workflow considerations, the thermodilution CO monitor was calibrated once prior to the beginning of data collection for each animal, which may have contributed to the slight discrepancies between devices during the post bleeding phase. Nevertheless, given the high level of accuracy between the wearable device and the SG measurements, further studies should investigate whether these findings are also translatable to humans particularly under conditions of hemodynamic instability.

From a clinical standpoint, an important advantage of the wearable device is its reliance on PPG technology, which is widely used in medical care. This technology allows easy patient monitoring without the need for extensive training of care providers. On the basis of our results, it appears that implementation of this wearable device in humans would allow reliable, yet safer, monitoring of hospitalized patients who may be in critical, unstable conditions similar to the hemorrhagic shock tested in our study or other life-threatening situations such as sepsis, anaphylaxis or cardiogenic shock. Alongside in-hospital intensive care, such a device might enable better monitoring, triage, and treatment in prehospital scenarios, including prolonged field care and natural disaster zones with limited access. Moreover, the current SARS-CoV-2 pandemic illustrated that frequent, non-invasive and wireless means for advanced hemodynamic monitoring is important for proper management of large numbers of isolated patients without compromising the health of the medical staff or quality of treatment^33^. Thus, although the results from this study are promising, future studies should test the capabilities of the PPG-based technology in humans under these specific indications.

In conclusion, there is a clear need for reliable non-invasive technology capable of advanced hemodynamic monitoring. In this animal model of hemorrhagic shock, we report that measurements obtained from a novel PPG-based device offer a high level of reliability compared to existing standard invasive techniques, even in circumstances of moderate to severe and unstable hemorrhagic shock. Pursuant to further trials, such a device could offer reliable and straightforward monitoring capabilities in a variety of clinical settings, from various hospital departments to pre-hospital and ambulatory settings. Most importantly, this novel PPG-based technology enables continuous non-invasive monitoring and timely focused care, which may minimize morbidity and mortality without compromising measurement accuracy.

## Methods

### Animals

Eleven white domestic female pigs (Laboratory Animals Farm, Lahav, Israel; Table 1) were housed in the institutional animal facility accredited by the Association for Assessment and Accreditation of Laboratory Animal Care International (AAALAC). Water and normal appropriate diet were available ad libitum. The experimental procedures were performed 7 days after acclimatization. Food was withheld starting the night before the procedure.

### Study design

Animals were sedated and connected to various monitoring devices as described below. The animals were monitored for ~ 30 min (“pre-bleeding” phase) before 35% of their total blood volume was withdrawn (“bleeding phase”; Table 1). Animals were then monitored (“post-bleeding phase”) for up to 7 h (median of 447 ± 184 min) or until death if occurred earlier. No additional intervention was performed. Hemodynamic parameters were documented every 5 min until the end of bleeding (both pre- and bleeding phases) and every 20 min during the post-bleeding phase using the AL and the SG catheter. At the same time, measurements were recorded every 5 min using the PPG-based wearable device, attached either to the animals’ skin, tongue, or tail in order to achieve the best PPG signal readings. Despite the capability of the wearable device to allow a high rate of measurement, we included and analyzed only the PPG-based device data points that were recorded in parallel to the AL and SG catheter. At the end of the observation period, surviving animals were euthanized.

### Preparation and anesthesia

Animals were sedated with Xylazine (1 mg/kg, IM, Eurovet Animal Health BV, Netherlands), and anesthesia was induced with Ketamine (10 mg/kg, IM, Vétoquinol SA, France). The ear vein was then cannulated for intravenous administration of a mixture of Diazepam (2 mg, IV, TEVA Pharmaceutical Industries Ltd., Israel), Ketamine (400 mg, IV, Vétoquinol SA, France), Propofol (1–4 mg/kg, IV, Fresenius Kabi Austria Gmbh Austria), and Tramadol (5 mg/kg, IM, Rafa Laboratories Ltd. Israel) for analgesia. Cefazolin (1 g, IV, Panpharma SA, France) was given prophylactically. The pigs were then intubated with a cuffed silastic endotracheal tube (7.0-mm, Portex Tracheal Tube, UK). Anesthesia was maintained with 2% isoflurane (Piramal Critical Care Inc. PA, USA) in 100% oxygen, and animals were ventilated using controlled mechanical ventilation (Excel 210-SE anesthesia machine m-Datex-Ohmeda Inc, Madison, or Narkomed-2B Anesthesia Machine—North American Drager, USA). Tidal volume was set to 10 ml/kg with a respiratory rate of 13–15 breaths per minute adjusted to an end-tidal pressure of 35 mmHg CO_2_ (P_ET_CO_2_) at baseline. Arterial oxyhemoglobin saturation was continuously monitored and kept at ~ 98–100%.

### Surgical procedures

The following vessels were cannulated using the over-the-wire (Seldinger) technique with introducers (Cordis, Fremont, CA) inserted into: the left common carotid artery for invasive blood pressure monitoring and heart rate; the right internal jugular vein for venous blood sampling; pulmonary artery catheter placement (Swan-Ganz CCOmbo, Edwards Lifesciences, Irvine, CA); right femoral artery cannulation for arterial blood sampling and induction of bleeding. A urinary catheter (Tiemann Catheter 12F) was placed for collection and output monitoring. Body temperature was monitored rectally. Pulse oximeter was placed on the tail lingual or buccal surface to measure arterial oxyhemoglobin saturation. Continuous three-lead electrocardiogram monitoring was obtained using electrodes placed on the animal’s right forelimb, left forelimb, and left hind limb. At the end of the observation period, surviving animals were euthanized by an intravenous injection of KCl solution (Fagron Group BV, Rotterdam, Netherlands).

### Hemorrhagic shock

Hemorrhage was induced by the controlled bleeding of 35% of the animal’s calculated blood volume^34^. Blood was withdrawn manually from the right femoral artery by using a syringe in 50 ml aliquots. The rate of bleeding was controlled to keep mean arterial pressure from dropping below 30 mmHg. When necessary, bleeding was stopped, and the animal was allowed to recover prior to the resumption of bleeding. The total bleeding time was kept between 30 and 60 min.

### Hemodynamic measurements

SBP, DBP, HR, and CO were monitored and recorded every 5 min up to the end of bleeding and every 20 min thereafter. Pulmonary artery pressures were monitored using a Datex-Ohmeda Cardiocap 5 (Datex-Ohmeda Inc, Madison, WI). CO was monitored using a Vigilance II Monitor (Edwards Lifesciences, Irvine, CA).

### Photoplethysmography-based monitoring device

**Figure 4:**
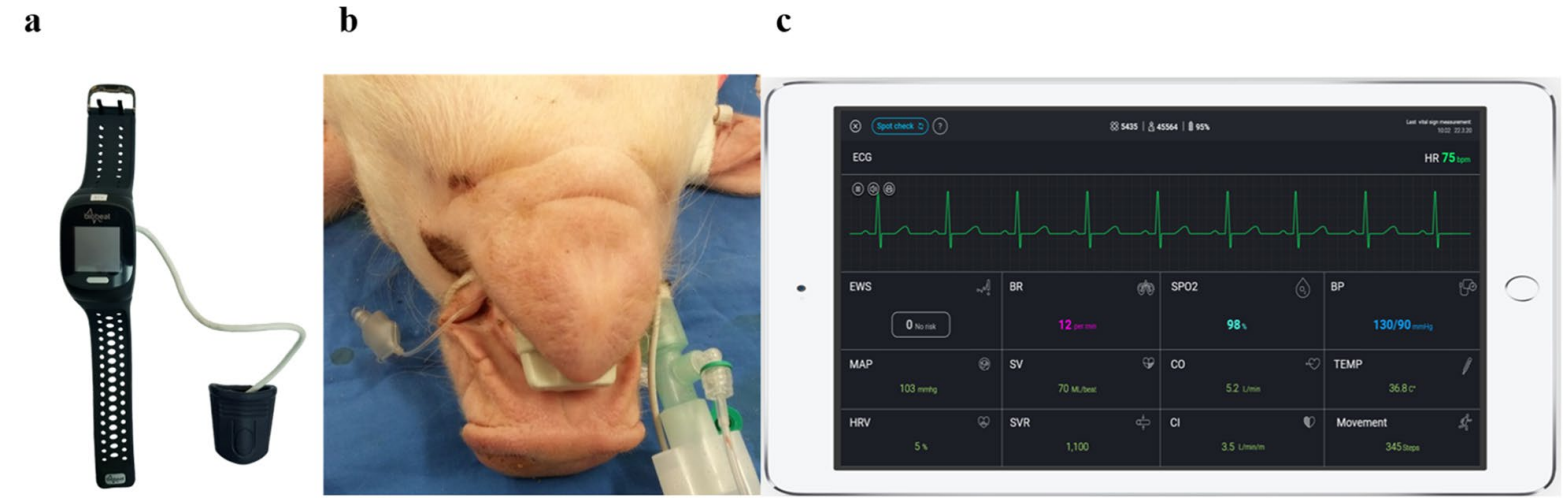
The PPG-based device: (**a**) The wristwatch configuration adapted to the swine study by attaching it to a tongue or tail connector. (**b**) The device attached to the tongue of a ventilated swine. (**c**) The device is wirelessly connected to a tablet using Bluetooth. The figure demonstrates the graphic user interface of the device’s application.

PPG is commonly applied for pulse oximetry, usually transmitting light in specific red and infrared wavelengths which is absorbed by a detector on the other side of the tissue (hence attached to relatively thin body parts such as fingers, ear lobes, etc.). While passing through the tissue, and upon interaction with oxy- and deoxyhemoglobin, these wavelengths show a unique absorbance pattern. The detector can measure the changing absorbance at each of the wavelengths, and by that it can determine the absorbance resulting from the pulsating arterial blood. The currently used device is based on reflective PPG (Fig. 4), in which the transmitted light is partially reflected from the tissue and detected by a photodiode detector positioned near the light source transmitter. The high resolution of the PPG wave combined with advanced algorithms allows the sensor to capture minute changes as well as tracking numerous vital signs derived from the pulse contours, including advanced hemodynamic parameters such as BP, HR, CO, and more. Tracking the changes of blood pressure is achieved after a pre-set baseline calibration process as described below, and is based on Pulse Wave Transit Time (PWTT) technology combined with Pulse Wave Analysis (PWA). The algorithm used was not trained or adapted using the data acquired in this study, rather it was trained using data from the public MIMIC Critical Care Database source (https://mimic.physionet.org/; https://doi.org/10.1038/sdata.2016.35), and from numerous clinical studies conducted earlier by the company. The baseline calibration measurement is by a simple offset using either an approved non-invasive, cuff-based device or an invasive (AL) device as in the case of this study, with the average value of three consecutive measurements entered into the device's management application, and from that moment on it tracks changes in blood pressure. Calibration is needed once every 3 months, which increases its clinical usability. Within the context of this study, calibration was conducted only once after the animals were sedated, ventilated, and the instrumentations (including the AL and SG catheters) were inserted.

### Data processing and analysis

Data obtained from the AL and SG catheter for SBP, DBP, HR, and CO were screened for outliers according to the following criteria: initially, percentage differences (%∆) between each value and the preceding and proceeding values were calculated. For each variable separately, the average standard deviation (SD) for the sum of preceding and proceeding %∆ was then obtained. Next, the individual data points (%∆) were screened, and any value for which the percentage difference from both the previous and next values was > ± 2SD was eliminated. In total, 6 out of 1312 (< 0.5%; HR: *n* = 3; SBP: *n* = 1; DBP: *n* = 1; CO: *n* = 1) outlier values were excluded from the final analysis.

### Statistics

Statistical analyses were performed separately for each of the three phases (i.e., pre-bleeding, bleeding, post-bleeding) and for all phases combined. The Kolmogorov–Smirnov and Shapiro–Wilk tests were used to assess normality, as these tests are sensitive to outliers. To define the degree of correlation between the two methods (i.e. invasive vs. PPG-based), the linear regression formula was defined, Pearson’s correlation coefficient was calculated, and the hypothesis that the slope and intercept are equal to zero was tested. The level of absolute agreement between AL/SG measurements and those obtained from the wearable device for SBP, DBP, HR, and CO was evaluated using intraclass correlation coefficients (ICC) and standard error of the mean (SEM) as well as Bland–Altman plots. Results of the Bland–Altman analyses are reported as mean biases ± 95% limits of agreement (LOA). All other results are presented as means ± SD. Due to the unstable hemodynamic state imposed on the animals and to better assess the PPG device’s ability to track *changes* (between successive measurement points) or trends in BP and CO, four quadrant plots analyses were performed. Concordance rate was calculated as the percentage of data points lying in the upper right and lower left quadrants relative to all points. To reduce statistical noise, we implemented an exclusion zone of ± 2 mmHg and 0.5 l/min for BP and CO measurements, respectively, as suggested by Critchley et al.^35^ and Saugel et al.^36^. Statistical analyses were considered significant if *p* < 0.05. Data were analyzed using SPSS 23.0 (SPSS Inc., Chicago, IL, USA).

### Study approval

This study conformed to the Guide for the Care and Use of Laboratory Animals (National Academy Press, Washington, D.C. 1996). Animal care and experimental procedures were approved by the Ethics Committee of The Hebrew University Faculty of Medicine, Jerusalem, Israel (MD-13-13751-3).
